# Supplementary material for: A yield and cost comparison of tuberculosis contact investigation and intensified case finding in Uganda
Source: PLoS One. 2020 Jun 8;15(6):e0234418. doi: 10.1371/journal.pone.0234418 (PMC7279581; doi:10.1371/journal.pone.0234418)
Supplement: S1 File — (PDF) [file pone.0234418.s001.pdf]

Ministry of Health

Intensified TB Case Finding Guide

Use the guide to identify presumptive TB:

In HIV Clinic, OPD, IPD and Congregate settings

This guide should be administered by either a health care provider or lay provider at the health facility

STEP 1: The person conducting the assessment asks the following questions:

1.	Has the patient been coughing for 2 weeks or more? ***(for known HIV patients assess cough regardless of duration)***	**Yes**	**No**
2.	Has the patient had persistent fevers for 2 weeks or more?	**Yes**	**No**
3.	Has the patient had noticeable weight loss (more than 3 kg)	**Yes**	**No**
4.	Has the patient had excessive night sweats for 3 weeks or more? *(**for adults**)*	**Yes**	**No**
5.	Has the child had poor weight gain in the last one month*? ***(ask for children < 5 years)***	**Yes**	**No**
6.	Has the child had contact with a person with Pulmonary Tuberculosis or chronic cough? ***(ask for children < 5 years)***	**Yes**	**No**

***poor weight gain** (Weight loss, **or** very low weight (weight-for-age less than –3 z-score), **or** underweight (weight-for age less than –2 z-score), **or** confirmed weight loss (>5%) since the last visit, **or** growth curve flattening)*

STEP 2: Guide for Actions to take

- If **yes to question 1** request for sputum test and refer to clinician for further investigations. ***Direct the patient to a designated area for people with chronic cough.***
- **If no to question 1 and yes to any other question;** refer to clinician for further investigations
- If **no to all questions:** repeat TB Assessment at subsequent visits

For Children who are unable to produce sputum, refer to clinician for further investigations

STEP 3: Record of Information at Health facility level

1. If you are in a clinic attending to patients enrolled in HIV care record this information on the comprehensive ART card; this information should then be transferred to the Pre ART or ART register.

2. If you are in a clinic setting (not attending to patients enrolled in HIV care e.g. OPD) and presumptive TB case is found, record the information in a presumptive TB register.

JULY 2013 EDITION
